# Robustness of Radiomics in Pre-Surgical Computer Tomography of Non-Small-Cell Lung Cancer

**DOI:** 10.3390/jpm13010083

**Published:** 2022-12-29

**Authors:** Maria Paola Belfiore, Mario Sansone, Riccardo Monti, Stefano Marrone, Roberta Fusco, Valerio Nardone, Roberto Grassi, Alfonso Reginelli

**Affiliations:** 1 Departement of Precision Medicine, Campania University “Luigi Vanvitelli”, 80138 Naples, Italy; 2 Department of Electrical Engineering and Information Technology, University ‘Federico II’, 80131 Naples, Italy; 3 Department of Research & Development IGEA Span, 80013 Carpi, Italy

**Keywords:** radiomic, lung cancer, radiogenomic, CT, texture analysis

## Abstract

**Background:** Radiomic features are increasingly used in CT of NSCLC. However, their robustness with respect to segmentation variability has not yet been demonstrated. The aim of this study was to assess radiomic features agreement across three kinds of segmentation. **Methods**: We retrospectively included 48 patients suffering from NSCLC who underwent pre-surgery CT. Two expert radiologists in consensus manually delineated three 3D-ROIs on each patient. To assess robustness for each feature, the intra-class correlation coefficient (ICC) across segmentations was evaluated. The ‘sensitivity’ of ICC upon some parameters affecting features computation (such as bin-width for first-order features and pixel-distances for second-order features) was also evaluated. Moreover, an assessment with respect to interpolator and isotropic resolution was also performed. **Results**: Our results indicate that ‘shape’ features tend to have excellent agreement (ICC > 0.9) across segmentations; moreover, they have approximately zero sensitivity to other parameters. ‘First-order’ features are in general sensitive to parameters variation; however, a few of them showed excellent agreement and low sensitivity (below 0.1) with respect to bin-width and pixel-distance. Similarly, a few second-order features showed excellent agreement and low sensitivity. **Conclusions**: Our results suggest that a limited number of radiomic features can achieve a high level of reproducibility in CT of NSCLC.

## 1. Introduction

Radiomics is gaining increasing interest as a tool for objective and quantitative radiological image evaluation [1,2,3,4,5,6,7,8,9,10,11,12,13,14,15,16]; radiomic features have also been recently used in relation to genomic data (radiogenomics) [11,12,17,18,19,20,21,22]. However, in spite of the claimed ‘objectivity’, there is still an operator-dependent component in radiomic features. In particular, it has been observed that radiomic features are dependent on image perturbation [1]; moreover, radiomic features reproducibility might depend on region-of-interest (ROI) segmentation performed by different physicians.

A number of studies have reported on radiomics reproducibility. For example, in [3], the authors focused on reproducibility across different computerised tomography (CT) equipment or reconstruction techniques. However, they used only one segmentation obtained in consensus by three radiologists which might not be available in clinical routine. Many studies [1,2,3,4,23,24,25,26,27,28,29,30,31] highlighted that from a clinical point of view, before radiomic features can be introduced into the routine clinical evaluation of non-small-cell lung cancer (NSCLC) patients, robustness with respect to segmentation variability must also be assessed [31,32,33,34].

In this study, we evaluated the robustness of radiomic features across segmentations and evaluated the influence of other parameters (specifically, bin-width, pixel-distance in second-order features, interpolator and isotropic resolution) on a set of CT volumes from patients suffering from NSCLC.

## 2. Methods

### 2.1. Patients

In this retrospective study, we enrolled 48 patients who underwent high resolution CT (HRCT) for NSCLC evaluation before surgery.

### 2.2. Image Acquisition

Images were acquired with one single scanner at our institution with standard reconstruction kernel. Equipment data are reported in Table 1. For every patient, a high-resolution chest CT (GE Revolution 128 MDCT) without contrast was carried out on the patient in the supine position to determine the position, the dimension and number of the lesion. The protocol of the HRCT for every patient was acquired with thin sections defined as <1.5, rotation time 200–500 ms, matrix size 512 × 512, collimation: 1.5–3 mm, slice thickness < 1.5 mm and FOV 35 cm. Reconstruction algorithm: high spatial frequency. Every protocol was acquired in full inspiration.

### 2.3. ROIs Delineation

Two expert radiologists in consensus (MB and RM) manually delineated three segmentations on each patient. The software used was ITK-snap [35,36]. The first segmentation was free-hand and was delineated as accurately as possible. The second segmentation was a free-hand rough one. The third was delineated using a rough polygonal. The second and third segmentations were drawn including the lesion and some tissues around it.

### 2.4. Features Extraction

Radiomic features might support radiologists in quantifying medical image characteristics. However, given the difficulties of standardisation in this field, recently [4] the Imaging Biomarker Standardisation Initiative (IBSI) has assessed reproducibility. Therefore, we used radiomic features that have been approved by the IBSI committee.

Specifically, Pyradiomics [22] is an open-source python library allowing the calculation of radiomic features compatible and approved within the IBSI. In this study, we used Pyradiomics version 3.0.1.

We computed the following categories of radiomic features: shape 3D, first-order and second-order (glcm, glrlm, glszm, ngtdm and gldm). Each feature has been computed on original and wavelet (coif1) images. See [4,22] for a detailed account for features formulas and interpretation. A total of 851 features were computed.

In order to assess the dependence on important parameters used for features computation, we computed features using several values of bin-width and pixel-distance. Bin-width is the gray-level bin-width of the histogram and it can affect mainly features which require histogram computation: gray-level co-occurrence matrix (GLCM) and gray-level run length matrix. We repeated the feature computation for bin-width = 10, 20 and 40. Pixel-distance specifies the distances between the centre voxel and the neighbourhood, for which angles should be generated; it affects mainly the gray-level co-occurrence matrix (GLCM) and neighbouring gray-tone difference matrix (NTGDM). We repeated the feature computation using distance = 1, 2 and 4 pixels.

Moreover, we assessed robustness also with respect to isotropic resolution and type of interpolator. In particular, we used nearest-neighbour, linear and spline interpolators as the default implemented in pyradiomics. Resolutions were fixed at 1, 2 and 2.5 mm.

### 2.5. Robustness Evaluation

In order to quantify ‘robustness’ of a feature across segmentations, we used the intra-class correlation coefficient (ICC). The ICC is described in [37]. In particular, per each feature, we computed the lower bound of the 95% confidence interval for ICC. As a reference value for robustness, in line with other studies [3,4] we used ICC = 0.9, which indicates ‘excellent’ agreement. The computation of ICC and subsequent statistical analysis was performed in R version 3.6.3 [38] using package ‘irr’ version 0.84.1.

**Figure 1:**
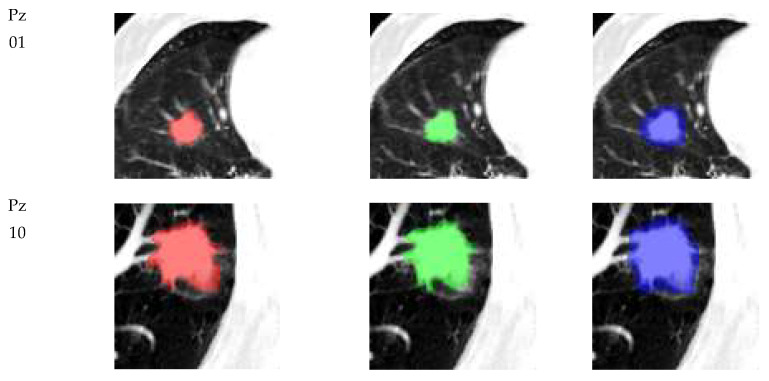

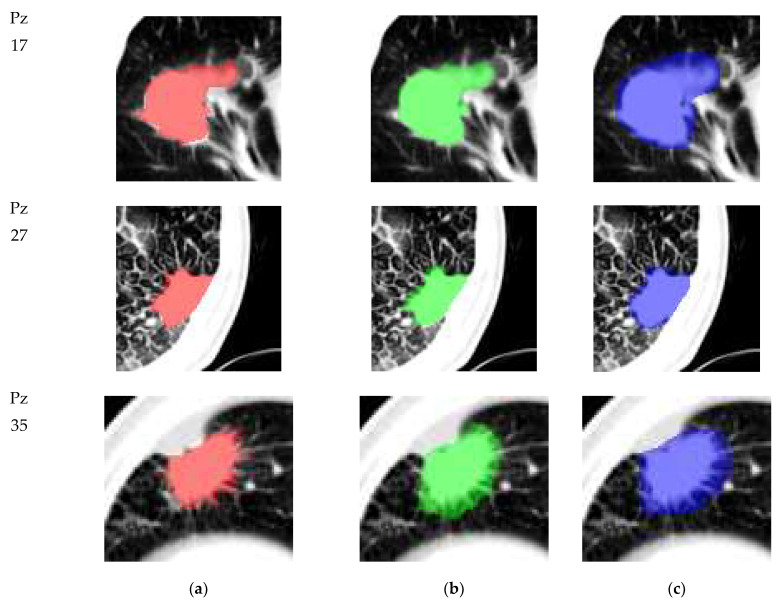
Illustrative examples of manual segmentations. For each patient on a row, different segmentations are showed superimposed on CT data: (**a**) polygonal, (**b**) free-hand accurate and (**c**) free-hand rough.

As regards to the ‘sensitivity’ to other parameters (bin-width, pixel-distances, type of interpolator and isotropic resolution), it was assessed per each parameter within each feature using the range of variation of ICC having other fixed parameters. In lack of previous studies, we arbitrarily considered a variation below 0.1 as low sensitivity. In Figure 1 there is a graphical summary of our analysis.

Lastly, the correlation between each feature reproducibility and lesion size (shape feature Voxel Volume) was analysed. To do this kind of analysis, it was not possible to use ICC (because patient information are flattened); we instead used the coefficient of variation (CV) which is the ratio between a measure of dispersion (standard deviation) of the distribution of one feature over a patient and the average value of the same feature over the same patient. The correlation between CV and tumour size is then computed.

## 3. Results

**Scheme 1:**
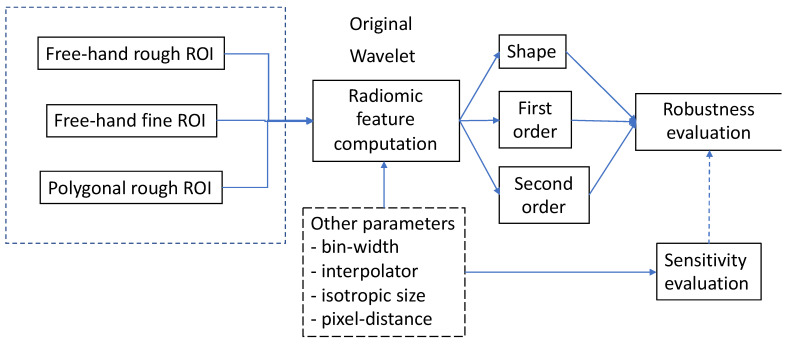
Flowchart of the method used in this study to evaluate robustness of radiomic features. Three 3D ROIs have been delineated on each subject included in the study. Radiomic features from the original image and from the wavelet transformed have been computed using different values of four parameters: bin-width, pixel-distance, interpolator and isotropic resolution. ICC has been used for robustness evaluation and variability of ICC across a parameter was used as an index of ‘sensitivity’ of the feature to the parameter.

Patient demographic data and lesion type information are presented in Table 2. Figure 1 reports some illustrative examples of ROIs delineated on five randomly chosen patients. Flowchart of the method used in this study to evaluate robustness of radiomic features is shown in Scheme 1.

**Figure 2:**
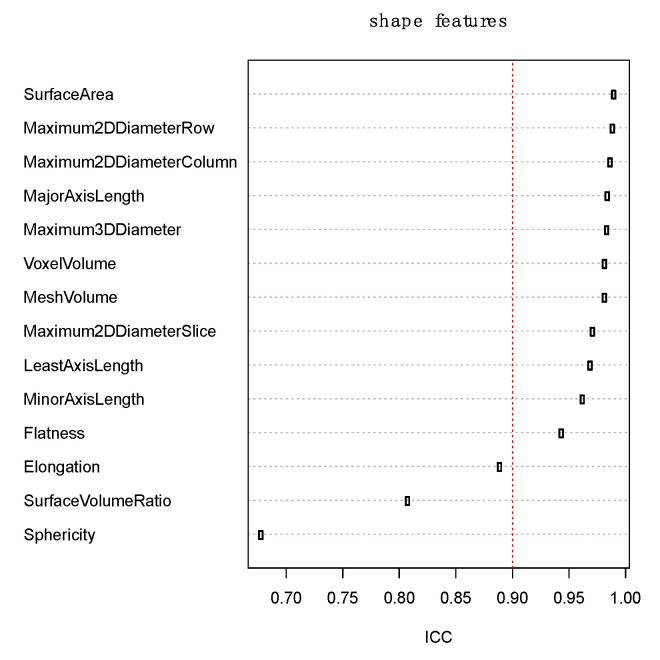
Robustness of shape features across segmentations. The vertical dashed red line indicates the ICC > 0.9 threshold. Pyradiomics provides 14 shape features. The robustness is above 0.60 for all of them. However, three of them are less robust (<0.90) across segmentations (Sphericity, SurfaceVolumeRatio and Elongation); this might be because they largely depend on the precise ROI contours. They do not depend on bin-width, distance, resolution or interpolator. With the exception of Flatness, the other features have a robustness greater than 0.95.

**Figure 3:**
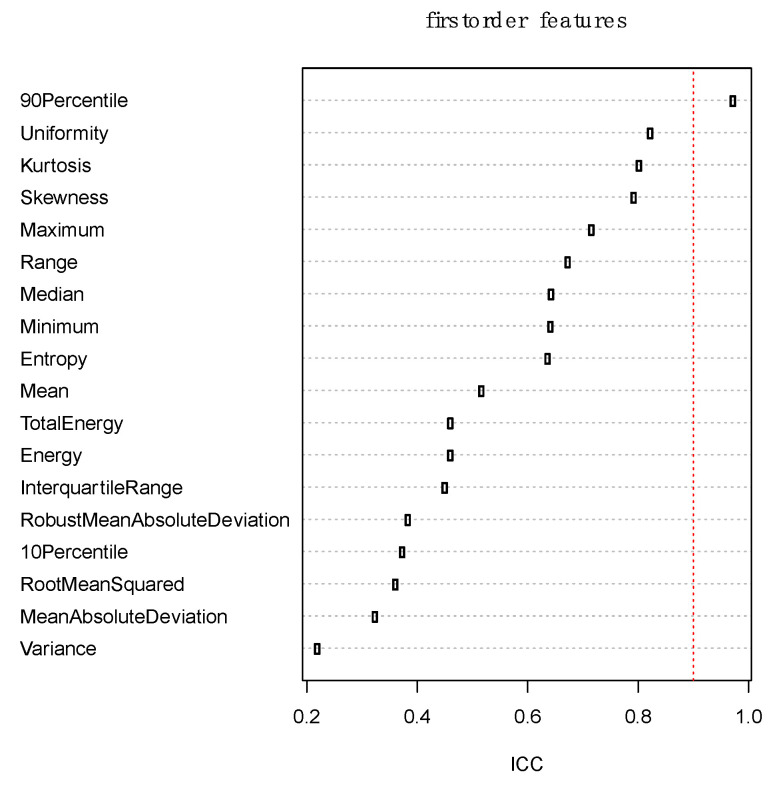
Robustness of first-order histogram features across segmentations. The vertical dashed red line indicates the ICC > 0.9 threshold. Pyradiomics provides 18 first-order features. The robustness (ICC) is above 0.8 only for skewness, kurtosis, uniformity and 90 percentile. The latter has ICC > 0.90. They are not dependent on bin-width (only entropy has slight dependence). Because they are first-order, they are not dependent on distance, neither do they depend on resolution or interpolator.

**Figure 4:**
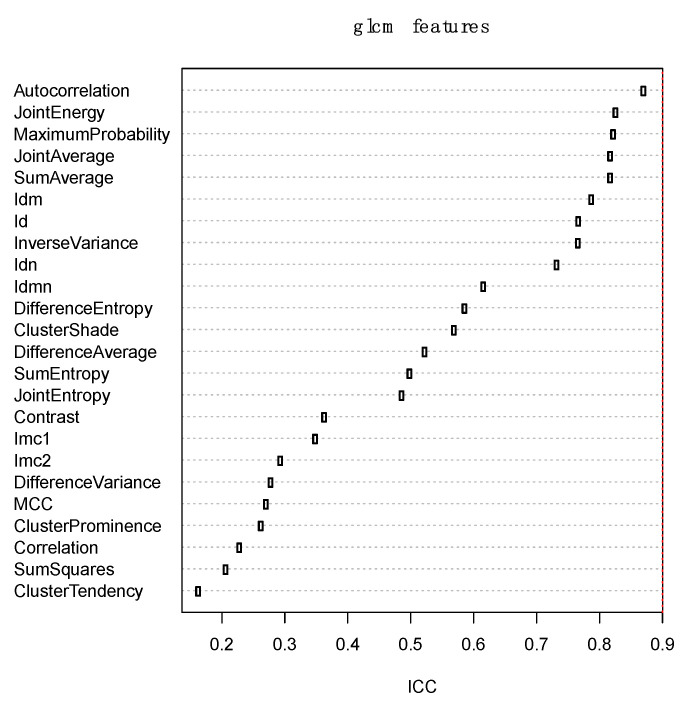
Robustness of second-order GLCM features across segmentations. Pyradiomics computes 24 second-order GLCM features. The robustness (ICC) is above 0.8 per all values of distance, only for Autocorrelation, Idn, Idmn, SumAverage, JointAverage, InverseVariance, JointEnergy, MaximumProbability, Id and Idm. Robustness of many features across segmentations is dependent on bin-width and distance (the vertical dashed red line on the right indicates the 0.9 threshold).

**Figure 5:**
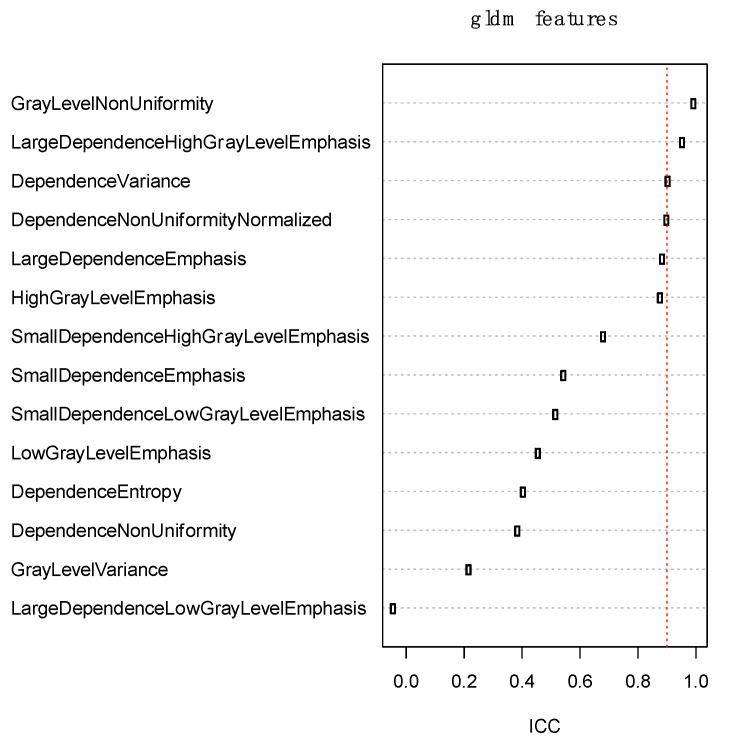
Robustness of second-order GLDM features across segmentations. Pyradiomics computes 14 second-order GLDM features. The robustness (ICC) is above 0.8, per all values of distance and binw, only for nine of them. Robustness of many features across segmentations is dependent on bin-width and distance (the vertical dashed red line indicates the 0.9 threshold).

**Figure 6:**
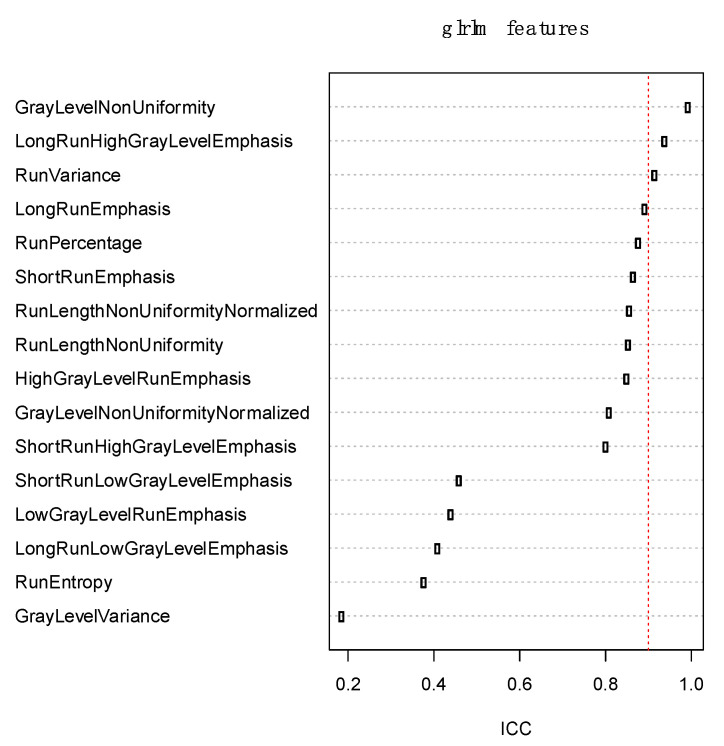
Robustness of second-order GLRLM features across segmentations. Pyradiomics computes 16 second-order GLRLM features. The robustness (ICC) is above 0.8, per all values of distance and binw, only for 11 of them. Robustness of many features across segmentations is dependent on bin-width but not on distance (the vertical dashed red line indicates the 0.9 threshold).

**Figure 7:**
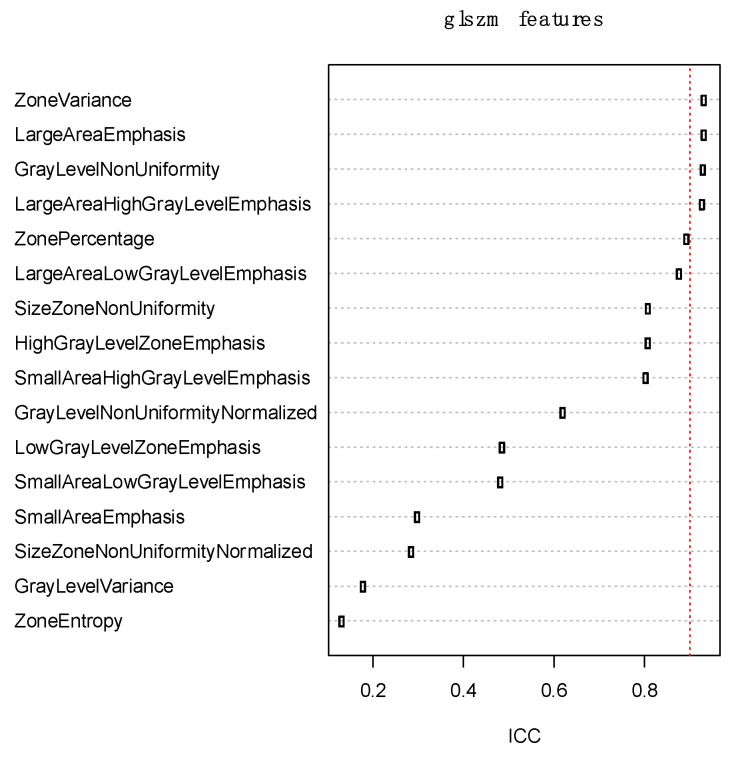
Robustness of second-order GLSZM features across segmentations. Pyradiomics computes 16 second-order GLSZM features. The robustness (ICC) is above 0.8, per all values of distance and binw, only for 11 of them. Robustness of many features across segmentations is dependent on bin-width but not on distance (the vertical dashed red line indicates the 0.9 threshold).

**Figure 8:**
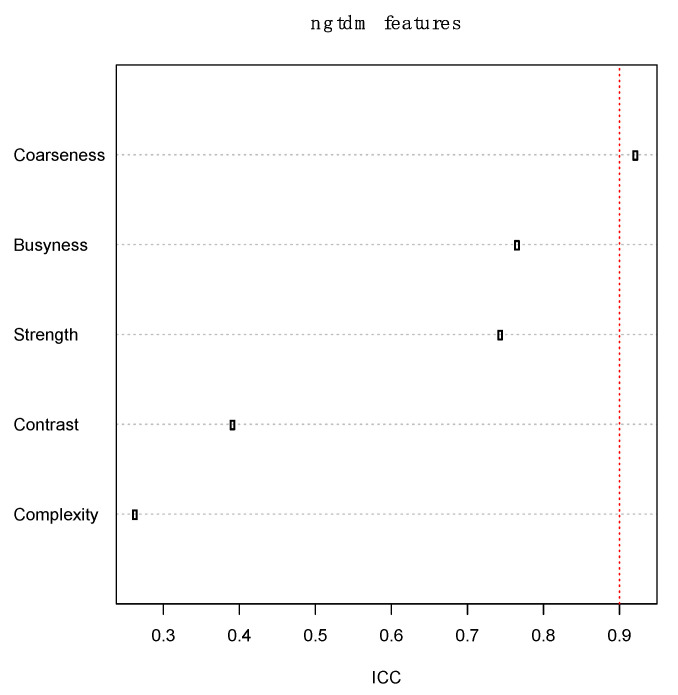
Robustness of second-order NGTDM features across segmentations. Pyradiomics computes five second-order NGTDM features. The robustness (ICC) is above 0.9, for all values of distance and binw, only for one of them. Robustness of many features across segmentations is dependent on bin-width but not on distance (the vertical dashed red line indicates the 0.9 threshold).

**Figure 9:**
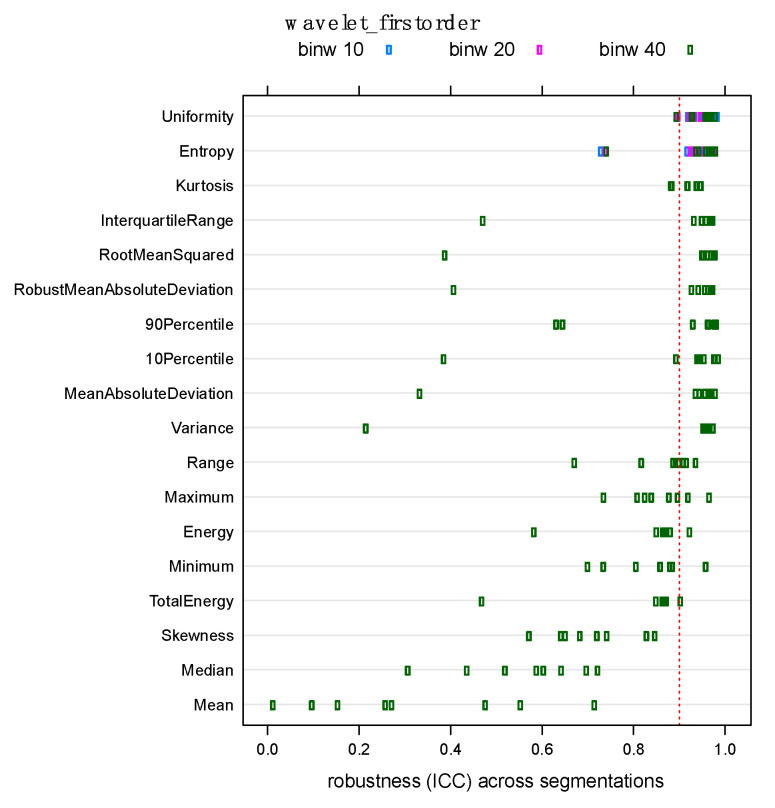
Robustness of wavelet-based first-order histogram features across segmentations. The vertical dashed red line indicates the ICC > 0.9 threshold. Pyradiomics provides 18 first-order features. The robustness (ICC) is above 0.8 only for Skewness, Kurtosis, Uniformity and 90Percentile. The latter has ICC > 0.90. They are not much dependent on bin-width (only entropy and uniformity have slight dependence). Because they are first-order, they are not dependent on distance neither do they depend on resolution or interpolator. However, the wavelet features are dependent on the direction used for wavelet computation (each circle on a line is a different gradient direction).

**Figure 10:**
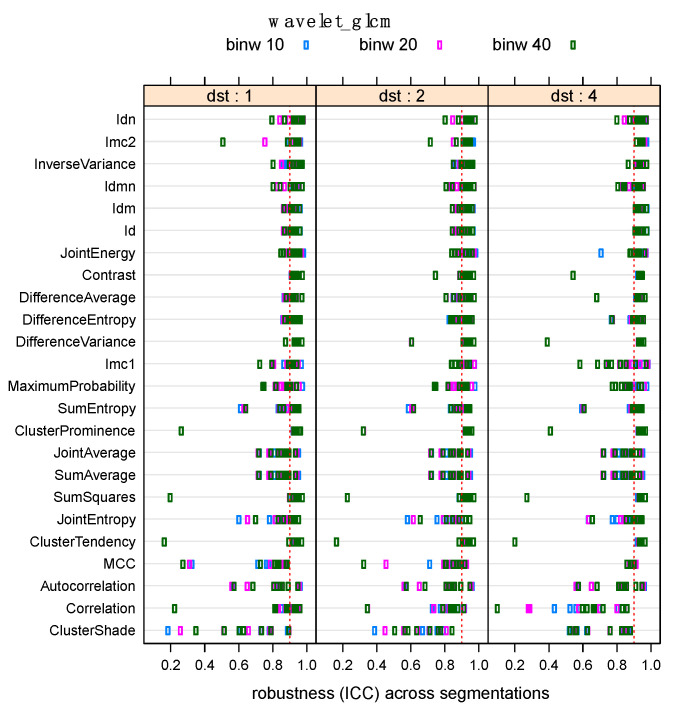
Robustness of wavelet-based second-order GLCM features across segmentations. The vertical dashed red line indicates the ICC > 0.9 threshold. Pyradiomics provides 24 such features. The robustness (ICC) is above 0.9 only for few of them. They are dependent on bin-width (binw: 10, 20, 40) and on (dst: 1, 2, 4). Because they are second-order, they are dependent on distance (dst) but they do not depend much on resolution or interpolator. However, the wavelet features are dependent on the direction used for wavelet computation (each circle on a line is a different gradient direction).

**Figure 11:**
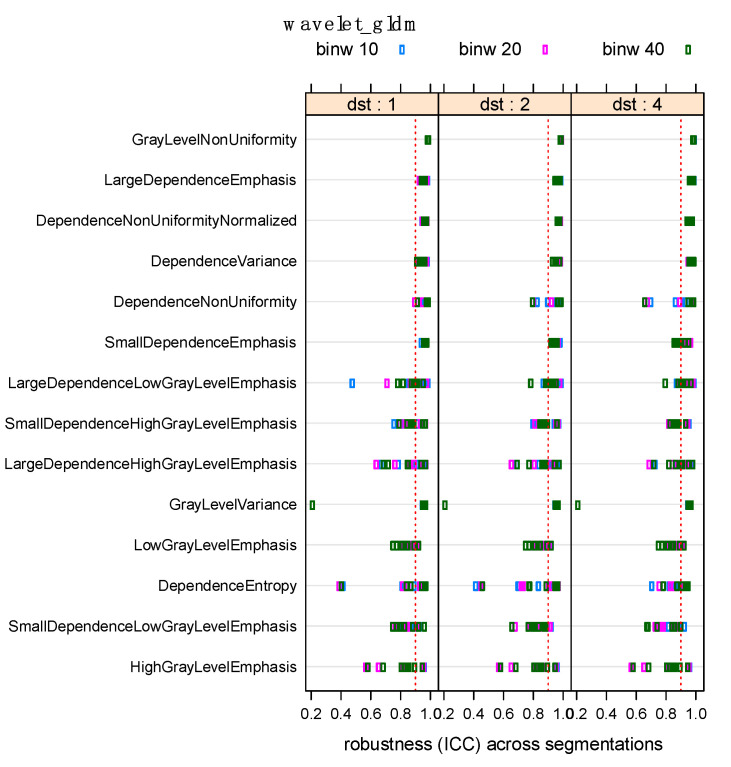
Robustness of wavelet-based second-order GLDM features across segmentations. The vertical dashed red line indicates the ICC > 0.9 threshold. Pyradiomics provides 14 such features. The robustness (ICC) is above 0.9 only for few of them. They are dependent on bin-width (binw: 10, 20, 40) and on (dst: 1, 2, 4). Because they are second-order, they are dependent on distance (dst) but they do not depend much on resolution or interpolator. However, the wavelet features are dependent on the direction used for wavelet computation (each circle on a line is a different gradient direction).

**Figure 12:**
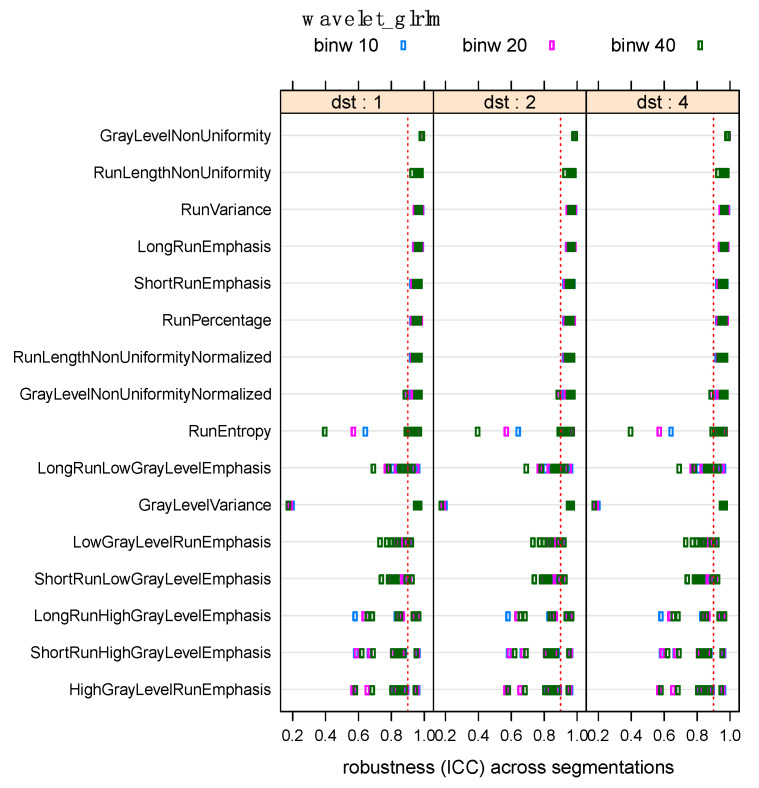
Robustness of wavelet-based second-order GLRLM features across segmentations. The vertical dashed red line indicates the ICC > 0.9 threshold. Pyradiomics provides 16 such features. The robustness (ICC) is above 0.9 only for few of them. They are dependent on bin-width (binw: 10, 20, 40) and on (dst: 1, 2, 4). Because they are second-order, they are dependent on distance (dst) but they do not depend much on resolution or interpolator. However, the wavelet features are dependent on the direction used for wavelet computation (each circle on a line is a different gradient direction).

**Figure 13:**
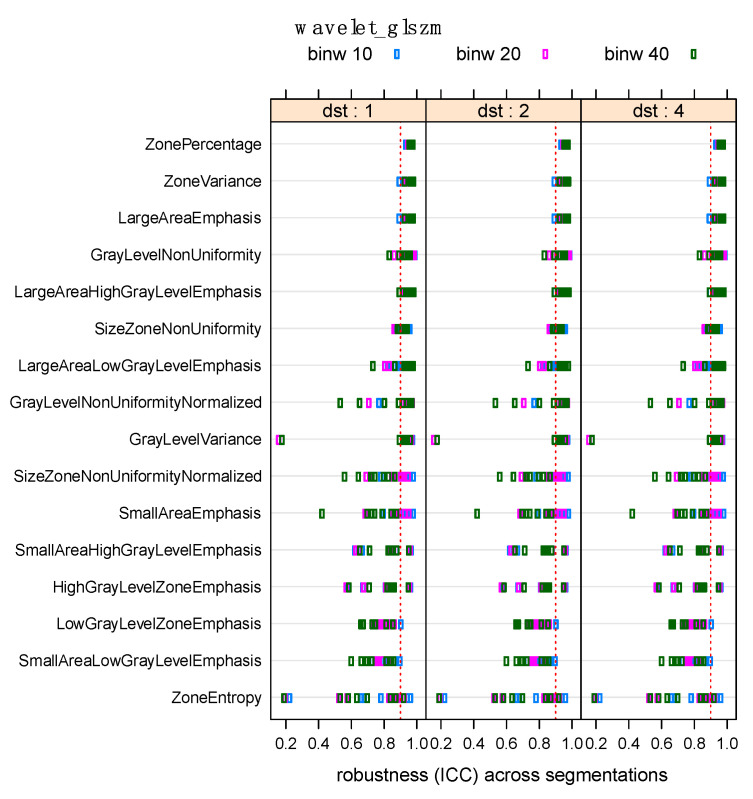
Robustness of wavelet-based second-order GLSZM features across segmentations. The vertical dashed red line indicates the ICC > 0.9 threshold. Pyradiomics provides 16 such features. The robustness (ICC) is above 0.9 only for few of them. They are dependent on bin-width (binw: 10, 20, 40) and on (dst: 1, 2, 4). Because they are second-order, they are dependent on distance (dst) but they do not depend much on resolution or interpolator. However, the wavelet features are dependent on the direction used for wavelet computation (each circle on a line is a different gradient direction).

**Figure 14:**
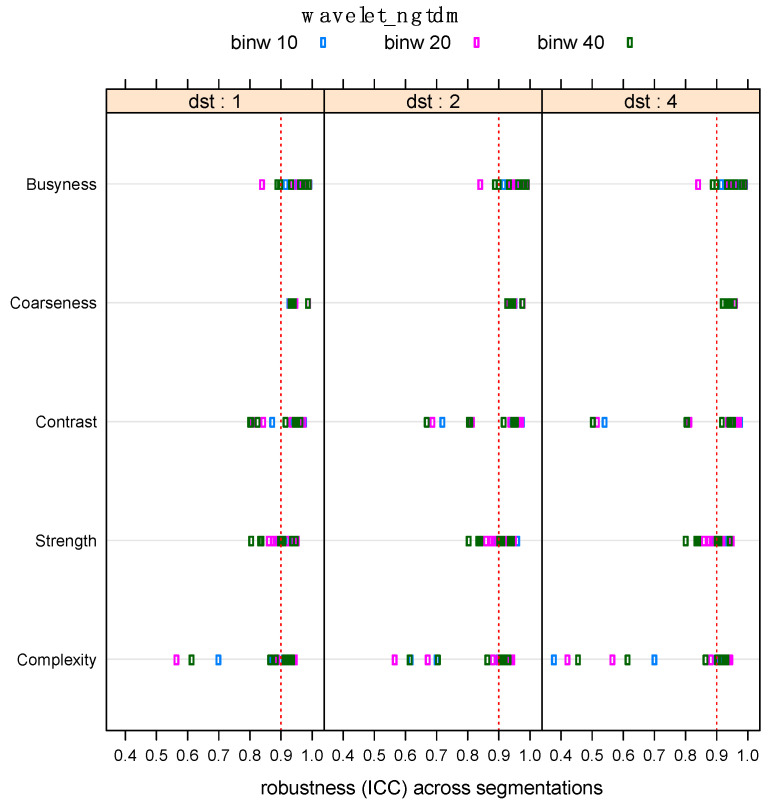
Robustness of wavelet-based second-order NGTDM features across segmentations. The vertical dashed red line indicates the ICC > 0.9 threshold. Pyradiomics provides five such features. The robustness (ICC) is above 0.9 only for few of them. They are dependent on bin-width (binw: 10, 20, 40) and on (dst: 1, 2, 4). Because they are second-order, they are dependent on distance (dst) but they do not depend much on resolution or interpolator. However, the wavelet features are dependent on the direction used for wavelet computation (each circle on a line is a different gradient direction).

Figure 2, Figure 3, Figure 4, Figure 5, Figure 6, Figure 7, Figure 8, Figure 9, Figure 10, Figure 11, Figure 12, Figure 13 and Figure 14 report the main results of our study. In particular, for each feature, we reported the ICC value with respect to the three segmentations. We grouped robustness per category of features. In particular, we considered ‘shape’, ‘first-order’, ’second-order-original’ and ‘second-order-wavelet’. As an example, we comment in detail on Figure 2; we report robustness (ICC) evaluated on shape features. These features (shape) seem to have generally high robustness. It is not less than 0.6 and many of them have robustness higher than 0.9; only three of them have an ICC below 0.9. This kind of feature does not depend on bin-width, distance, resolution or interpolator.

We can observe similar behaviours for other features (Figure 3, Figure 4, Figure 5, Figure 6, Figure 7, Figure 8, Figure 9, Figure 10, Figure 11, Figure 12, Figure 13 and Figure 14). However, in some cases, the ICC can be well below 0.9 indicating poor robustness of that feature. Moreover, in most wavelet-based and second-order features, there is a dependence on bin-width, distance, resolution or interpolator.

**Figure 15:**
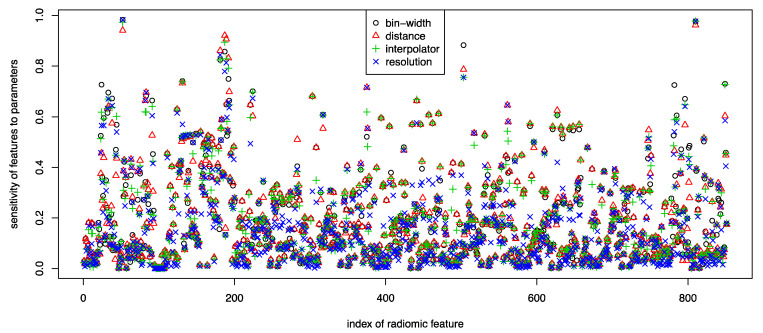
Sensitivity to other parameters. Per each feature, the range of variation of ICC is reported per each parameter (bin-width, pixel distance, interpolator and isotropic resolution) having fixed the other three.

As regards to sensitivity of the ICC of features to other parameters such as bin-width, pixel-distance, isotropic resolution and interpolator, we report in Figure 15 the sensitivity per each feature. The first 107 features were computed from the original image; the other 744 were computed from wavelet images. The index in the figure refers to a feature list given as a separate file. We note that ‘shape’ features (index from 94 to 107) have very small (approximately zero) sensitivity.

**Figure 16:**
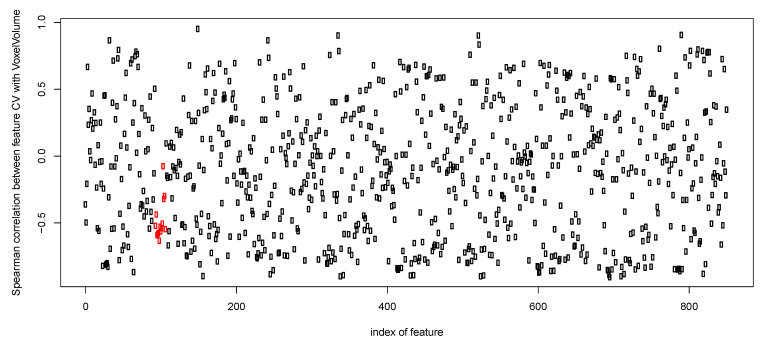
Correlation between CV for each feature (on *x*-axis) and lesion size (shape feature Voxel Volume). Spearman method was used. Red circles indicate correlation for ‘shape’ features; only a weak correlation exists.

Figure 16 reports the correlation between each feature CV and lesion size. The Spearman method was used. Red circles represent correlations with ‘shape’ features; only a weak correlation exists.

## 4. Discussion

The aim of this study was to evaluate the robustness of radiomic features with respect to ROI segmentation in the case of pre-surgical CT of NSCLC. We quantified robustness as ‘agreement’ among three segmentations using the intra-class correlation coefficient (ICC). We chose ICC = 0.9 as a reference value indicating an ‘excellent’ agreement [37]. The sensitivity of ICC upon bin-width, distance, resolution or interpolator was also evaluated. We assessed the following feature categories: shape (14 features), first-order (18), gray-level co-occurrence matrix (24), GLDM (14), GLRLM (16), GLSZM (16) and NGTDM (5): a total of 107 features. Moreover, the same features were assessed on wavelet images (coif1) using High/Low filtering in x-y-z directions for a total of 851 features.

Our results showed that some features were robust having high ICC; however, a number of features were sensitive to segmentation, having low ICC. Moreover, a dependence on bin-width, distance, resolution or interpolator has been observed for many features.

Most importantly, we observed that shape features showed high robustness (ICC > 0.90). This is in line with intuition, as shape features (e.g., VoxelVolume, maximum diameter or surface area) should not vary too much across segmentations. It should be noticed, however, that surface-volume ratio and sphericity can reach very low ICC values (down to 0.75).

Another important result of our study is that we observed a low number of features robust with respect to segmentations and to bin-width, distance, resolution or interpolator. In fact, our results showed that a large number of first- and second-order features have an ICC lower than 0.9. For these features the agreement ICC, across segmentations and other source of variability, is less than excellent.

Lastly, the correlation analysis showed that ‘shape’ features CV is only weakly negatively correlated with lesion size: as tumour size increases, CV weakly decreases. This might be explained by the fact that the larger the tumour size, the larger the segmentation variability by manual delineation. Moreover, Figure 16 shows that there is a large variability among feature CV vs lesion size.

Assessment of radiomics robustness is an important task for guaranteeing reproducibility of radiomic analysis across segmentations; it has been also addressed in other papers [3,4]. A very important paper in this field is [4] in which a large number of radiomics features was evaluated for reproducibility across 25 teams. However, they used only one CT of a patient suffering from lung cancer. Subsequently, in [3], the authors evaluated radiomics reproducibility of NSCLC across different CT equipment and reconstruction algorithms. In this last paper, the authors analysed 103 patients. However, the authors did not explicitly address the dependence on segmentations and feature parameters.

The results of our study are in line with previous studies [3,4,13,15,23,25,26,30,39,40,41,42,43,44,45,46,47]; however, as a difference, we specifically addressed segmentation variability with a fixed CT device but analysed the dependence on bin-width, distance, resolution or interpolator.

Commentary is in order regarding the applicability of our results in clinical settings. The fact that a feature might be suitable for clinical application should be assessed, not only looking for high reproducibility; in fact, a certain variability of a feature might be tolerated given that the variability in the patients is larger. Further, in ultimate analysis, features are used as an input to classifiers; therefore, also the specific combination of classifier plus feature must be optimised. Specifically, our study addressed the problem of features selection only from the point of view of high reproducibility but did not address the points just mentioned.

One important limitation of this study is the reduced sample size (48 patients). In order to gain insight into the reproducibility of radiomics features, large databases should be analysed.

## 5. Conclusions

Radiomics analysis has the potential for improving diagnosis in many pathologies and in particular in NSCLC. However, the reproducibility of features with respect to variability in 3D ROI segmentations and other sources of variability in feature computation (such as bin-width, pixel-distance, interpolator and isotropic resolution) must be assessed before introduction into the clinical practice. In our study, we observed that ‘excellent’ agreement (ICC > 0.9) across different sources of variability can be achieved by a relatively small number of features: in particular, ‘shape’ features, some second-order features and some wavelet-based features.

## Figures and Tables

**Table 1:** CT equipment and acquisition parameters.

Pixel spacing	0.578–0.976 mm
Slice thickness	1.5 mm
KVP	120 kV
Equipment	Revolution HD—GE MEDICAL SYSTEMS
Scan options	Helical Mode

**Table 2:** Patient characteristics.

Sex	14 F, 34 M
Age	49–87 (mean 70, std 10)
Diagnosis	32 Adenocarcinoma, 14 Squamous, 1 Neuroendocrine 1 atypical carcinoid

## Data Availability

Image and segmentations data might be shared upon reasonable request.
